# Polymorphisms of Glucose-Regulated Protein 78 and Risk of Colorectal Cancer: A Case-Control Study in Southwest China

**DOI:** 10.1371/journal.pone.0066791

**Published:** 2013-06-20

**Authors:** Dan Zhang, Bin Zhou, Yuan Li, Mojin Wang, Cun Wang, Zongguang Zhou, Xiaofeng Sun

**Affiliations:** 1 Department of Gastrointestinal Surgery, West China Hospital, Sichuan University, Chengdu, Sichuan Province, China; 2 Laboratory of Digestive Surgery, West China Hospital, Sichuan University, Chengdu, Sichuan Province, China; 3 Division of Oncology, Department of Clinical and Experimental Medicine, Faculty of Health Sciences, County Council of Östergötland, University of Linköping, Linköping, Sweden; Centro di Riferimento Oncologico, IRCCS National Cancer Institute, Italy

## Abstract

Glucose-regulated protein 78 (GRP78), an endoplasmic reticulum chaperone, up-regulation serves as an efficient mechanism to promote malignant transformation of colorectal cancer (CRC) and protect CRC cells against apoptosis. Recently, the analysis of GRP78 polymorphisms has already determined that GRP78 rs391957 polymorphism could predict clinical outcome in CRC patients. Thus, we tested whether GRP78 polymorphisms are related to the risk of CRC. In this study, we detected two GRP78 polymorphisms (rs391957 (C>T) and rs430397 (G>A)) in 414 CRC cases and 502 hospital-based cancer-free healthy controls in Southwest China using a polymerase chain reaction–restriction fragment length polymorphism technique. Compared with the CC genotype, carriers of CT and TT genotypes of rs391957 polymorphism had higher risks of CRC (odds ratio (OR) = 1.39, 95% confidence interval (CI) = 1.06–1.83 for CT genotype and OR = 2.10, 95% CI = 1.06–4.14 for TT genotype, respectively). In CRC cases, the variant T allele was significantly associated with tumor invasion stage (P = 0.030), but not with status of lymph nodes metastasis (P = 0.052). Compared with the GG genotype, carriers of GA and AA genotypes of rs430397 polymorphism had higher risks of CRC (OR = 1.63, 95% CI = 1.23–2.15 for GA genotype and OR = 2.92, 95% CI = 1.23–6.94 for AA genotype, respectively). The rs430397 polymorphism was not associated with the clinicopathological characteristics of CRC. These data provide the first evidence that GRP78 rs391957 and rs430397 polymorphisms could serve as markers to predict the risk of CRC.

## Introduction

Colorectal cancer (CRC) is the third most common cancer worldwide, accounting for approximately 10% of total cancer cases. Almost 60% of the CRC cases occur in the developed countries [1]. However, the incidence of CRC is increasing in most of the developing countries, whereas trends are either stabilizing or declining in most of the developed countries [2]. As a developing country, China has experienced a two- to four- fold increase in incidence of CRC in recent decades [3]. In addition, the Chinese Hans have significantly higher incidence of CRC than other ethnic groups in Asia [4]. The pathogenesis of CRC is not completely understood but it is surely a multistep carcinogenic process in which genetic and epigenetic alterations accumulate sequentially [5]. Gene polymorphisms as genetic variations have been studied already in more than 35 different genes, and for several genes, association with CRC are observed [6], [7].

Glucose-regulated protein 78 (GRP78), also referred to as immunoglobulin heavy chain binding protein, is a member of the heat shock protein 70 family which is constitutively expressed and resides primarily in the endoplasmic reticulum (ER) [8], [9]. As a major ER chaperone, GRP78 facilitates protein folding and assembly, translocates newly synthesized polypeptides across the ER membrane, prevents intermediates from aggregating, targets misfolded proteins for proteasome degradation, binds Ca^2+^ and serves as a sensor for ER stress [10]–[12]. Moreover, overexpression of GRP78 can be dramatically induced by ER stress including glucose deprivation, disturbance of Ca^2+^ homeostasis, oxidative stress and hypoxia, and then triggers the unfolded protein response (UPR). UPR is a complex and multifaceted signal-transduction cascade and serves to limit the accumulation of unfolded proteins, contributing to limiting the damage in cells exposed to ER stress and protecting cells against death [9], [12]–[15].

Cancer cells are often confronted with hypoxia and glucose deprivation, thereby under the condition of ER stress with overexpression of GRP78. Recently, overexpression of GRP78 has been detected in several cancers such as brain cancer, breast cancer, lung cancer, prostate cancer, gastric cancer and CRC. Additionally, GRP78 has been shown to be associated with the development and progression of cancers. [16]–[23]. There were evidences to indicate that GRP78 polymorphisms, alone or in combination, had effect on the expression of GRP78 [24], [25]. Previous reports found that GRP78 rs430397 polymorphism was associated with the risk and prognosis of hepatocelluar cancer (HCC) and the prognosis of non-small cell lung cancer (NSCLC) [26], [27]. Meanwhile, it was also known that GRP78 rs391957 polymorphism might serve as a potential prognostic determinant for gastric and colorectal cancers [28]. The recent study showed GRP78 rs391957 polymorphism was associated with the risk of HCC, but the result remained contradictive [29]. Currently, little is known about the relationship of GRP78 polymorphisms and susceptibility to CRC, especially in the Chinese Han population. Therefore, we hypothesized that GRP78 polymorphisms might also affect the risk of CRC.

To test the hypothesis, we conducted a case-control study in a Chinese Han population to investigate whether GRP78 polymorphisms were associated with the risk and clinicopathological characteristics of CRC.

## Materials and Methods

### Study Subjects

A total of 414 CRC cases and 502 cancer-free controls were recruited for this case-control study. The patients were selected sequentially from January 2009 to August 2009 at the department of Gastrointestinal Surgery, West China Hospital, Sichuan University. All the cases had newly diagnosed, untreated primary CRC, who were identified by preoperative colonoscopy and CT scan, intraoperative exploration and postoperative pathological examination. The rectal cancer was regarded as the tumor located within 15 cm distance from the anal, and the non-rectal colon cancer was regarded as the tumor located beyond 15 cm distance from the anal. Patients with other cancer history and previous radiotherapy and chemotherapy were excluded. All the selected cases agreed to participate. The hospital-based controls were randomly selected from the same hospital during routine health checkups, who were cancer-free healthy individuals identified by colonoscopy and CT scan. The control group matched the case group in gender and age. All the participants were unrelated Chinese Hans in Southwest China. Written informed consent, blood samples and clinical data were collected from all the participants according to the protocols approved by the Ethics Committee of West China Hospital of Sichuan University.

### DNA Extraction and Genotyping

Genomic DNA was extracted from whole blood of each participant by conventional phenol/chloroform procedure. The concentration and purity of DNA were measured with a spectrophotometer. The isolated DNA was dissolved in TE buffer and stored in the refrigerator at −20°C before analysis. Two GRP78 polymorphisms (rs391957 (C>T) and rs430397 (G>A)) were genotyped using polymerase chain reaction (PCR)–restriction fragment length polymorphism technique. Briefly, forward and reverse primers were used for PCR amplification, and then the amplified products were digested with restriction endonucleases. Furthermore, the lengths of the PCR products and the digested fragments were determined by electrophoresis on 1.5% agarose gel and 3% agarose gel, separately. To genotype GRP78 rs391957 polymorphism, PCR primers were 5′-atctctcctgcgacttctga-3′ (forward) and 5′-gatggaggaagggagaacaa-3′ (reverse), and the size of amplified products was 167 bp. The PCR products were then digested with restriction endonuclease MboII. The variant T allele had the MboII restriction site and two bands (134 bp and 32 bp) were generated after the digestion, whereas the wild C allele lacked the restriction site and a single band (167 bp) was obtained. To genotype GRP78 rs430397 polymorphism, PCR primers were 5′-aattcaggacattgcatcta-3′ (forward) and 5′-tggacagcagcaccatac-3′ (reverse), and the size of amplified products was 271 bp. The PCR products were then digested with restriction endonuclease Hin1III. The variant A allele lacked the Hin1III restriction site and a single band (271 bp) was obtained, whereas the wild G allele had the restriction site and two bands (218 bp and 52 bp) were generated after the digestion. In addition, genotyping results were validated by direct DNA sequencing in a random 5% of samples for quality control. Genotype concordance was 100%.

### Statistical Analysis

Calculations for the power and sample size of our case-control design were carried out using PS program software. Results indicated that the number of recruited samples could provide adequate statistical power. Deviation from the Hardy-Weinberg equilibrium for each polymorphism was tested using chi-square test among the controls. The differences in demographic characteristics (e.g. gender and age) between the CRC cases and controls were compared using chi-square and t tests. The associations of genotypes distribution of the GRP78 polymorphisms with clinicopathological characteristics of the CRC cases were evaluated using chi-square test. Risk estimates were calculated for the co-dominant and dominant genetic models using the most common homozygous genotype as the referent category. The effects of genotypes of the GRP78 polymorphisms on the risk of CRC were represented as the odds ratios (ORs) with 95% confidence intervals (CIs) using unconditional logistic regression model adjusted for gender and age. All statistical tests were two sided, and P<0.05 was considered significant. All statistical analyses were performed using the PASW Statistics 18 (SPSS Inc, Chicago, IL).

## Results

Of the estimated allele ORs of 1.38 and 1.60, our study with 414 CRC cases and 502 matched controls provided the statistical power of 0.80 and 0.95 at the nominal type I error rate of 0.05, respectively, indicating that our samples could provide adequate power in identifying the association of the risk of CRC with the two GRP78 polymorphisms. The observed genotypes frequency distribution of the two GRP78 polymorphisms in the controls did not show significant deviation from Hardy-Weinberg equilibrium (data not shown).

### Characteristics of Study Population

The demographic and clinical characteristics of the study participants were summarized in Table 1. The mean age were 59.0 (±13.2) years and 58.2 (±9.1) years in the cases and controls respectively. Most of the cases and controls were male, and in the cases the gender ratio (male/female) was 1.39∶1 while the ratio was 1.5∶1 in the controls. There was no significant difference between the cases and controls in terms of age (P = 0.295) and gender (P = 0.592), indicating that the matching for two groups was successful. Most of the CRC cases were in NCCN stage II and III (288/412, 69.9%). Tumors of most CRC cases were located in the rectum (310/414, 74.9%).

**Table 1:** Baseline demographic and clinical characteristics of CRC cases and controls.

Characteristic	Cases		Controls		P
	N = 414	Frequencies	N = 502	Frequencies	
**Age**					
Mean ± SD	59.0±13.2		58.2±9.1		0.295^a^
**Gender**					
Males	241	58.2%	301	60%	
Females	173	41.8%	201	40%	0.592^b^
**TNM Stage**					
I	73	17.7%			
II	149	36.2%			
III	139	33.7%			
IV	51	12.4%			
**Tumor stage**					
T1	34	8.3%			
T2	55	13.3%			
T3	80	19.4%			
T4	243	59%			
**Lymph node**					
Negative	236	57.3%			
Positive	176	42.7%			
**Metastasis**					
No	361	87.6%			
Yes	51	12.4%			
**Tumor site**					
Rectum	310	74.9%			
Colon	104	25.1%			
**Differentiation**					
Well	24	6%			
Moderate	247	61.6%			
Poor	130	32.4%			
**Growth pattern**					
Expansive	150	38.4%			
Infiltration	241	61.6%			

a P was computed using t test;

b P was computed using chi-square test.

Among 414 CRC cases, 2 cases had missing data on TNM stage, 13 cases had missing data on tumor differentiation and 23 cases had missing data on tumor growth pattern.

### Association of Clinicopathological Characteristics of the CRC Cases with the Two GRP78 Polymorphisms

Association analyses between genotypes distribution of the two GRP78 polymorphisms and clinicopathological characteristics of the CRC cases were shown in Table 2. Distribution of the variant-allele-carrying genotypes (CT+TT) or CC homozygous genotype of GRP78 rs391957 polymorphism were significantly associated with tumor invasion stage (T1, T2, T3 and T4 stage) in the CRC cases (P = 0.030). Among patients with the variant T allele, 9.0%, 10.1%, 24.9% and 56.0% were in T1, T2, T3 and T4 stage respectively, whereas 7.6%, 16.1%, 14.8% and 61.5% of the patients with the CC homozygous genotype were in T1, T2, T3 and T4 stage respectively. There was a higher trend that the variant T allele carriers had less proportion of lymph nodes metastasis (37.6%) compared with the carriers with CC homozygous genotype (47.1%), although the difference was not statistically significant (P = 0.052). Meanwhile, genotypes distribution of GRP78 rs391957 polymorphism was not associated with other clinicopathological data, such as TNM stage, distant metastasis, tumor site, tumor differentiation and tumor growth pattern in the CRC cases (P>0.05). In addition, there was also no association between distribution of the variant-allele-carrying genotypes (GA+AA) or GG homozygous genotype of GRP78 rs430397 polymorphism and all the clinicopathological characteristics in the CRC cases (P>0.05).

**Table 2:** Association of genotypes of GRP78 polymorphisms with clinicopathological characteristics of the CRC cases.

Characteristic (No. of cases)	GRP78 rs391957		P^a^	GRP78 rs430397		P^a^
	CC (%)	CT+TT (%)		GG (%)	GA+AA (%)	
**Age**						
<59 (187)	102 (45.7)	85 (44.5)		116 (47.5)	71 (41.8)	
≥59 (227)	121 (54.3)	106 (55.5)	0.801	128 (52.5)	99 (58.2)	0.245
**Gender**						
Males (241)	131 (58.7)	110 (57.6)		139 (57)	102 (60)	
Females (173)	92 (41.3)	81 (42.4)	0.831	105 (43)	68 (40)	0.538
**TNM Stage**						
I (73)	42 (18.8)	31 (16.4)		42 (17.4)	31 (18.2)	
II (149)	71 (31.8)	78 (41.3)		91 (37.6)	58 (34.1)	
III (139)	81 (36.3)	58 (30.7)		82 (33.9)	57 (33.5)	
IV (51)	29 (13.1)	22 (11.6)	0.264	27 (11.1)	24 (14.2)	0.780
**Tumor stage**						
T1 (34)	17 (7.6)	17 (9.0)		17 (7.0)	17 (10.0)	
T2 (55)	36 (16.1)	19 (10.1)		32 (13.2)	23 (13.5)	
T3 (80)	33 (14.8)	47 (24.9)		50 (20.7)	30 (17.6)	
T4 (243)	137 (61.5)	106 (56.0)	0.030	143 (59.1)	100 (58.8)	0.672
**Lymph node**						
Negative (236)	118 (52.9)	118 (62.4)		143 (59.1)	93 (54.7)	
Positive (176)	105 (47.1)	71 (37.6)	0.052	99 (40.9)	77 (45.3)	0.376
**Metastasis**						
No (361)	194 (87)	167 (88.4)		215 (88.8)	146 (85.9)	
Yes (51)	29 (13)	22 (11.6)	0.675	27 (11.2)	24 (14.1)	0.369
**Tumor site**						
Rectum (310)	168 (75.3)	142 (74.3)		182 (74.5)	128 (75.3)	
Colon (104)	55 (24.7)	49 (25.7)	0.817	62 (25.5)	42 (24.7)	0.852
**Differentiation**						
Well (24)	11 (5.1)	13 (7.1)		14 (6.0)	10 (6.0)	
Moderate (247)	134 (61.7)	113 (61.4)		148 (63.5)	99 (58.9)	
Poor (130)	72 (33.2)	58 (31.5	0.688	71 (30.5)	59 (35.1)	0.611
**Growth pattern**						
Expansive (150)	83 (39.3)	67 (37.2)		88 (36.6)	67 (40.9)	
Infiltration (241)	128 (60.7)	113 (62.8)	0.668	144 (63.4)	97 (59.1)	0.389

a P was computed using chi-square test.

Among 414 CRC cases, 2 cases had missing data on TNM stage, 13 cases had missing data on tumor differentiation and 23 cases had missing data on tumor growth pattern.

### Genotypes of the Two GRP78 Polymorphisms were Associated with Increased Risk of CRC

Logistic regression analyses of genotypes of the two GRP78 polymorphisms both showed significant differences between the CRC cases and controls in Table 3. Compared with the CC homozygous genotype of GRP78 rs391957 polymorphism, the CT heterozygous (Adjusted OR = 1.39, 95% CI = 1.06–1.83, P = 0.018) and TT homozygous (Adjusted OR = 2.10, 95% CI = 1.06–4.14, P = 0.033) genotypes were both significantly associated with higher risk of CRC. The overall variant T allele carriers (CT+TT) also had significantly higher risk of CRC compared with the CC homozygous carriers (Adjusted OR = 1.45, 95% CI = 1.11–1.89, P = 0.006), suggesting that the variant T allele of GRP78 rs391957 polymorphism may be a deleterious allele. In addition, similar trend of higher risk of CRC was detected in analyses of genotypes of GRP78 rs430397 polymorphism. Compared with the GG homozygous genotype, the GA heterozygous (Adjusted OR = 1.63, 95% CI = 1.23–2.15, P = 0.001) and AA homozygous (Adjusted OR = 2.92, 95% CI = 1.23–6.94, P = 0.015) genotypes were both significantly associated with higher risk of CRC. The overall variant A allele carriers (GA+AA) also had significantly higher risk of CRC compared with the GG homozygous carriers (Adjusted OR = 1.70, 95% CI = 1.29–2.23, P<0.001), thus the variant A allele of GRP78 rs430397 polymorphism may be a deleterious allele as well.

**Table 3:** Adjusted odds ratios (ORs) and 95% confidence intervals (CIs) for CRC in relation to genotypes of GRP78 polymorphisms.

Genotype	Controls (%)	Cases (%)	OR^a^ (95% CI)	P
**GRP78 rs391957**				
CC	315 (63)	223 (54)	1	
CT	172 (34)	169 (41)	1.39 (1.06–1.83)	0.018
TT	15 (3)	22 (5)	2.10 (1.06–4.14)	0.033
CT+TT	187 (37)	191 (46)	1.45 (1.11–1.89)	0.006
**GRP78 rs430397**				
GG	356 (71)	244 (59)	1	
GA	138 (27)	154 (37)	1.63 (1.23–2.15)	0.001
AA	8 (2)	16 (4)	2.92 (1.23–6.94)	0.015
GA+AA	147 (29)	170 (41)	1.70 (1.29–2.23)	<0.001

a Adjusted for age and gender.

## Discussion

To our knowledge, this is the first study which has investigated whether GRP78 rs391957 and rs430397 polymorphisms are associated with the risk of CRC. In this hospital-based case-control study, we found that the CT heterozygous, TT homozygous and combined (CT+TT) genotypes of GRP78 rs391957 polymorphism and the GA heterozygous, AA homozygous and combined (GA+AA) genotypes of GRP78 rs430397 polymorphism were both significantly associated with higher risk of CRC in a Chinese Han population, suggesting that the two GRP78 polymorphisms probably play potential roles in the development of CRC.

In the USA, new cases with rectal cancer accounted for approximately 40% of cases with CRC [30]. However, we found that 75% CRCs occurred in the rectum in our cases, which was inconsistent with rectal cancer accounting for 40–50% in overall Han population. Our data was similar to that of the previous study about southwestern Chinese population by Wang *et al.* where 77% CRCs were rectal cancers in their cases [31]. One possible explanation is the incidence of cancer should be region-specific. Due to the vast territory and large population in China, life style and economic development are different in different regions, with different morbidities for different cancers. For this reason, now that our study focused on southwestern Han population in China, the high proportion of rectal cancer in our cases which might reveal the epidemiological situation of CRC in southwestern Han population was probably different from the proportion of rectal cancer in overall Han population. On the other hand, as southwest China is an economically underdeveloped area, the majority of people in southwest China are unable to afford routine screening for CRC. Therefore, most diagnosed cases were presented as symptomatic diseases. While rectal cancer may be more obvious than non-rectal colon cancer, patients with rectal cancer have a greater possibility of getting examination and treatment than those with non-rectal colon cancer. This may be another reason for high proportion of rectal cancer in our cases. Yet despite all this, the definitely proportions of rectal cancer and non-rectal cancer in southwestern Han population would need further investigation in large and multiple-centers studies. Additionally, the ratio of males to females with CRC in our study was 1.39∶1, which was also similar to that of 1.36∶1 in the previous study about southwestern Chinese population by Wang *et al.* [31]. Although our ratio in CRC was higher than that in the USA which is usually well balanced between males and females, we considered that the gender ration should also be region-specific because of differences in risk behaviors and ethnic groups. The previous study which showed the lower rates observed among females compared with males may be related to differences in risk behaviors associated with colorectal cancer, such as smoking, and the differing effect of obesity in men and women, also pointed at it [32]. Thus, the higher ratio of males to females with CRC might be the representative of gender ratio in southwestern Chinese population.

It is clear that overexpression of GRP78 occurs in various human cancers and cancer cell lines, correlating with malignancy, metastasis and poor prognosis [13]. The elevated expression of GRP78 increased the WHO pathologic grade of primary astrocytoma, which strongly predicted the patients’ prognosis [16]. The expression of GRP78 was up-regulated in prostate cancer cells compared with benign tissue, and the patients with higher expression of GRP78 had almost twice the risk of dying from prostate cancer compared with those with weak expression [20]. GRP78 was highly expressed in gastric cancer and appeared to be an independent survival predictor [21]. One previous study reported that overexpression of GRP78 appeared to correlate with histological severity from the normal colon tissue to colon adenoma to colon carcinoma, which indicated that overexpression of GRP78 might be a marker for malignant transformation of CRC [22]. Furthermore, another study showed that knockdown of GRP78 inhibited the proliferation of CRC cells and increased the apoptosis of CRC cells in vitro, which indicated that the expression of GRP78 might enhance the proliferation of CRC cells and protect them against apoptosis [23].

Several studies have demonstrated that the polymorphisms in the promoter of gene are likely to enhance or attenuate the expression of gene [33]–[35]. The rs391957 polymorphism is located in the promoter of GRP78 gene [24]. It has been noted that the rs391957 polymorphism altered the expression of GRP78 in cells under different conditions. Specifically, in normal cells, the variant T allele carriers had lower basal promoter activity than the CC homozygous carriers. However, in cells under the condition of ER stress, the variant T allele carriers had significantly higher expression of GRP78 than the CC homozygous carriers [25]. Thus, the variant T allele of GRP78 rs391957 polymorphism probably enhances the expression of GRP78 in various cancers thereby affecting the development and progression of cancers. These findings are consistent with the previous study which showed that the CRC patients with the CT heterozygous or TT homozygous genotypes had a significantly higher risk of tumor recurrence compared with the patients with the CC homozygous genotype [27]. Subsequently, our present study has similar findings, which showed that the variant T allele carriers had higher risk of CRC compared with the wild C allele carriers. Collectively, these data provide preliminary support that the variant T allele of GRP78 rs391957 polymorphism may be both a susceptible marker and a prognostic marker for CRC, and have an important function in the development and progression of CRC due to enhancing the expression of GRP78. However, some results still remain contradictive. The latest study showed that the C allele of GRP78 rs391957 polymorphism was associated with increased risk of HCC and the C allele carriers had higher mRNA and protein expression of GRP78 in HCC [29]. The possible explanation is that the conditions of ER stress are different in various types of cancer. Thus, more and further investigations are needed to discover and confirm the roles of GRP78 rs391957 polymorphism.

We also found that the variant T allele of GRP78 rs391957 polymorphism was associated with local tumor invasion of CRC. However, due to the retrospective design and relative numbers of cases involved, further prospective biomarker embedded clinical trials and cell and tissue experiments will be necessary to validate our result and identify what role the rs391957 polymorphism plays in progression of CRC. In our study, T4 stage was the majority of the cases. Tumor stage is often associated with lack of screening, access to medical care and so on. Thus, our result might also reveal that there is a lack of screening for CRC in southwestern Chinese population. Patients with CRC were unable to get prompt diagnosis, so that the majority of them were at advanced stages when they received proper treatment. It is necessary that southwestern Chinese pay more attention to routine screening for CRC. In addition, the previous study found higher expression of GRP78 contributed to increased lymph node metastasis in gastric cancer patients [21]. In contrast, we found the CRC patients with the variant T allele contributing to higher expression of GRP78 might have less possibility of lymph node metastasis. Limitations of our small sample size are likely to be the reason. Alternatively, the roles of GRP78 in affecting status of lymph node metastasis are different in various cancers.

The variant T allele frequency is approximately 29% in Asian population, 36% in European population, 29% in the USA and only 9% in African population. Thus the fact that most populations share similar proportions of T carriers reinforces the importance of our findings and the need for further study. However, ethnicity is of major importance in polymorphism analysis, and our findings also need studies about other ethnic populations to confirm it in the future.

The polymorphisms in the intron of gene have received less attention, but are beginning to be recognized for their potential contribution to the development and progression of diseases, including cancers [36]. There are splicing enhancer and splicing silencing sites throughout the introns of genes. Sequence alterations in any of these intronic sites can lead to alteration in splicing of the pre-mRNA which contributes to malignant progression [37]. The rs430397 polymorphism is just located in upstream from the intron/exon boundary within the fifth intron of GRP78 gene [36]. The previous study has firstly demonstrated that the variant A allele of GRP78 rs430397 polymorphism was associated with higher risk and poor prognosis of hepatocellular carcinoma [26]. Another study has also demonstrated that the AA homozygous carriers had higher RNA and protein expression of GRP78 compared with the GG homozygous carriers in tissues, and that the AA carriers had poor prognosis of non-small cell lung cancer [28]. The mechanism of GRP78 rs430397 polymorphism affecting the development and progression of cancer is unclear. One hypothesis is that this allele variation in the intron probably alters the splicing of the pre-mRNA, thereby affecting the expression of GRP78 by altering the efficiency of translation or mRNA stability [36]. The result of our study which showed that the variant A allele carriers had higher risk of CRC compared to the CC homozygous carriers has provided indirect evidences to support the hypothesis. Therefore, the variant A allele may be able to enhance the expression of GRP78, but the further function investigation is needed to explain the precise mechanism. Furthermore, the variant A allele of GRP78 rs430397 polymorphism may be a susceptible marker for CRC, but whether the variant A allele is associated with the prognosis of CRC is still not known.

There have been approximately 200 known polymorphisms within the GRP78 gene [36]. Based on previous studies, the polymorphisms of rs391957 and rs3216733 were completely linked [25], and the polymorphisms of rs391957, rs17840761 and rs12009 were in linkage disequilibrium while the haplotypes of them were not associated with CRC [28]. Furthermore, the polymorphisms of rs17840761 and rs12009 were not associated with CRC [28]. Additionally, it was not found that other polymorphisms and rs430397 polymorphism were in linkage disequilibrium.

### Conclusion

There were significant associations between the risk of CRC and the GRP78 rs391957 and rs430397 polymorphisms. Both the variant T allele of rs391957 polymorphism and the variant A allele of rs430397 polymorphism were associated with higher risk of CRC in southwestern Chinese Han population, though whether the variant T allele of rs391957 polymorphism affected local tumor invasion stages and status of lymph node metastasis needs to be further tested in prospective larger-scale studies in the future. These preliminary findings suggested that the GRP78 polymorphisms could provide clues to forecast susceptibility to CRC. But the precise mechanisms underlying the relationship of the GRP78 polymorphisms and CRC also need further investigation.
